# Integrated analysis of metabolic phenotypes in *Saccharomyces cerevisiae*

**DOI:** 10.1186/1471-2164-5-63

**Published:** 2004-09-08

**Authors:** Natalie C Duarte, Bernhard Ø Palsson, Pengcheng Fu

**Affiliations:** 1 Department of Bioengineering, University of California, San Diego, 9500 Gilman Drive, La Jolla, CA 92093-0412, USA; 2 Department of Molecular Biosciences & Bioengineering, University of Hawaii, 1955 East-West Road, Honolulu, HI 96822-2321, USA

## Abstract

**Background:**

The yeast *Saccharomyces cerevisiae *is an important microorganism for both industrial processes and scientific research. Consequently, there have been extensive efforts to characterize its cellular processes. In order to fully understand the relationship between yeast's genome and its physiology, the stockpiles of diverse biological data sets that describe its cellular components and phenotypic behavior must be integrated at the genome-scale. Genome-scale metabolic networks have been reconstructed for several microorganisms, including *S. cerevisiae*, and the properties of these networks have been successfully analyzed using a variety of constraint-based methods. Phenotypic phase plane analysis is a constraint-based method which provides a global view of how optimal growth rates are affected by changes in two environmental variables such as a carbon and an oxygen uptake rate. Some applications of phenotypic phase plane analysis include the study of optimal growth rates and of network capacity and function.

**Results:**

In this study, the *Saccharomyces cerevisiae *genome-scale metabolic network was used to formulate a phenotypic phase plane that displays the maximum allowable growth rate and distinct patterns of metabolic pathway utilization for all combinations of glucose and oxygen uptake rates. *In silico *predictions of growth rate and secretion rates and *in vivo *data for three separate growth conditions (aerobic glucose-limited, oxidative-fermentative, and microaerobic) were concordant.

**Conclusions:**

Taken together, this study examines the function and capacity of yeast's metabolic machinery and shows that the phenotypic phase plane can be used to accurately predict metabolic phenotypes and to interpret experimental data in the context of a genome-scale model.

## Background

The development of numerous high-throughput experimental techniques such as DNA microarrays, genome sequencing, and protein chips has revolutionized the analysis of biological systems and generated a catalog of information about a cell's components [1-3]. Efforts are now focused on the integration of this data to enable the systemic understanding of cellular functions [4-6]. This integration is typically in the form of a mathematical model that can be used to simulate complex cellular behaviors based on a limited amount of biological data.

Several modeling approaches have been implemented in the study of *Saccharomyces cerevisiae*. Flux-balance models of yeast have appeared for small-scale network reconstructions. Most of these studies are specific for growth conditions, such as anaerobic, glucose-limited metabolism [7], aerobic growth on galactose [8] or growth on mixtures of glucose and ethanol [9]. In addition to flux-balance models, many dynamic models of simplified central metabolic networks in yeast also have been developed [10,11], along with full-scale kinetic models specific to pathways such as glycolysis [12,13] and the pentose phosphate pathway [14]. These models have been useful to study detailed metabolic events such as concentration changes of individual metabolites and key flux splits.

Small-scale reconstructions can be limited in their prediction of cellular functions since these processes are typically dependent on the interaction of components at a whole-cell level. This has motivated the development of genome-scale models, several of which have already appeared for bacterial cells, including *Escherichia coli *[15,16], *Haemophilus influenzae *[17], and *Helicobacter pylori *[18]. We have previously reconstructed a genome-scale metabolic network of *Saccharomyces cerevisiae *based on its annotated genome sequence and a thorough examination of online pathway databases, biochemistry textbooks, and journal publications [19]. A total of 708 open reading frames, 1175 metabolic reactions, and 733 metabolites are accounted for in this stoichiometric network, which includes both cytosolic and mitochondrial compartments. This genome-scale reconstruction is the most comprehensive model of yeast metabolism to date and has been previously validated through *in silico *gene deletion studies [20] and the calculation of key physiological parameters [21].

The reconstruction and analysis of genome-scale microbial networks have advanced significantly in recent years [22,23], as has the development of a variety of constraint-based modeling methods that allow for the deduction a cell's phenotype based on its genotype and environmental conditions [24-28]. Phenotypic phase plane (PhPP) analysis is a constraint-based method used to obtain a global perspective of genotype-phenotype relationships in genome-scale metabolic networks. In PhPP analysis, flux balance analysis and linear programming are used to map all of the cellular growth conditions represented by two environmental variables onto a two-dimensional plane and identify phases with distinct metabolic pathway utilization patterns. Some applications of PhPP analysis include the study of optimal growth rates [29], adaptability of microorganisms [30,31], metabolic network functions and capacities [15], and the impact of gene regulations [32]. Thus, PhPP analysis provides a way to guide experiments and analyze phenotypic functions based on genome-scale metabolic networks.

The constraint-based modelling approach is based on the assumption that organisms have developed control structures to ensure optimal growth in response to environmental constraints [35]. Numerous experimental observations have been made in support of this hypothesis [36]. The mathematical descriptions for the PhPP have relevant metabolic meaning for the biological systems being studied. For example, any point in the PhPP corresponds to a single solution of the linear programming problem, which metabolically represents a possible growth behavior. A phase or region in the PhPP (where the shadow price is constant) represents a metabolic phenotype with specific pathway utilization. The shadow prices change continuously at the boundary from one phase to the next. Accordingly, the metabolic phenotype will vary. Metabolically, this is interpreted as a different optimal utilization of the metabolic pathways since each basis solution corresponds to a different flux distribution.

In this study, we formulate a glucose-oxygen phenotypic phase plane for yeast based on its recent genome-scale metabolic reconstruction [19]. The growth states predicted by the PhPP are then characterized using shadow price analysis, *in silico *gene deletion simulations, and *in vivo *growth experiments. Finally, we evaluate the network's predictions for these growth states by comparing *in silico *biomass formation and by-product secretion rates to *in vivo *measurements.

## Results

### *S. cerevisiae *phenotypic phase plane (PhPP)

**Figure 1:**
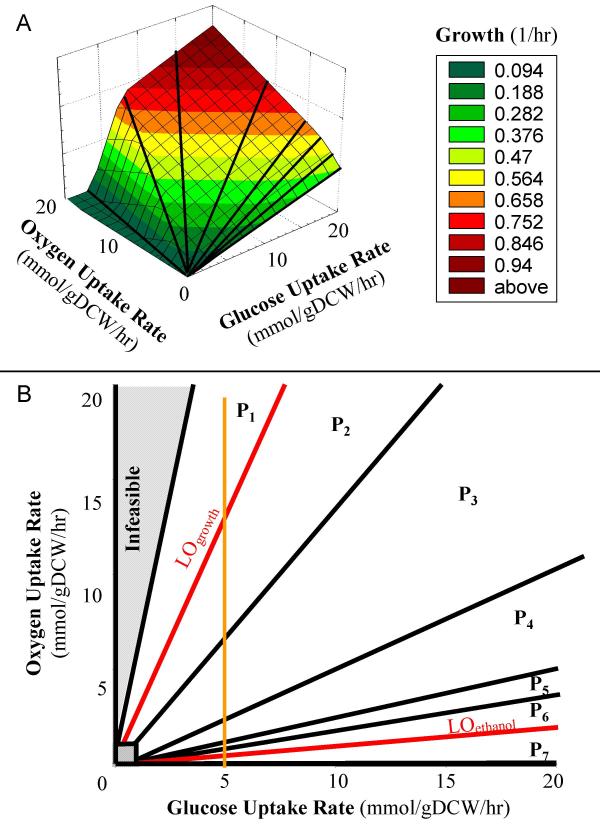
**The yeast glucose-oxygen phenotypic phase plane (PhPP). (a) **The three-dimensional *S. cerevisiae *PhPP drawn with Statistica™ (Statsoft, Tulsa, OK). The x and y axes represent the glucose uptake rate and oxygen uptake rate, respectively. The third dimension is the cellular growth rate. **(b) **A two-dimensional projection of the 3-D polytope in panel (a). The two lines of optimality are shown in red. LO_growth _represents optimal aerobic glucose-limited growth and LO_ethanol _corresponds to maximum ethanol production under microaerobic conditions. P_1 _- P_7 _represent phases with various metabolic phenotypes. The hatched regions correspond to infeasible growth conditions. The orange line (glucose uptake flux = 5 mmol/gDCW/hr) represents the conditions which were used for the simulations in Figure 2.

The *S. cerevisiae *genome-scale metabolic network constructed by Forster *et al. *[19] was used to generate a PhPP [33] that describes yeast's metabolic states at various levels of glucose and oxygen availability (Fig. 1). The surface of the three-dimensional PhPP corresponds to the maximum growth rate allowable for each pair of glucose and oxygen uptake rates in the x-y plane (Fig. 1a). All feasible metabolic flux distributions lie on or below this surface. The two-dimensional projection of the PhPP (Fig. 1b) has been divided into seven regions, or "phases," to allow for qualitative comparisons (P_1 _- P_7_). The seven phases represent areas of the PhPP that have distinct metabolic phenotypes as defined by shadow price analysis, which identifies how changes in metabolite levels affect biomass formation [33]. There also are two regions of the PhPP with infeasible steady-state flux distributions: the area along the *y*-axis and the small square near the origin. Growth is infeasible in the region between the ordinate and P1 since yeast cannot use more than six oxygen molecules per glucose molecule. The two red lines in Fig. 1b are lines of optimality (LO). LO_growth _represents optimal aerobic glucose-limited growth of *S. cerevisiae *in which substrates are completely oxidized to produce biomass and LO_ethanol _corresponds to maximum ethanol production under microaerobic conditions while growth is maximized.

### Simulation of optimal metabolic phenotypes

**Figure 2:**
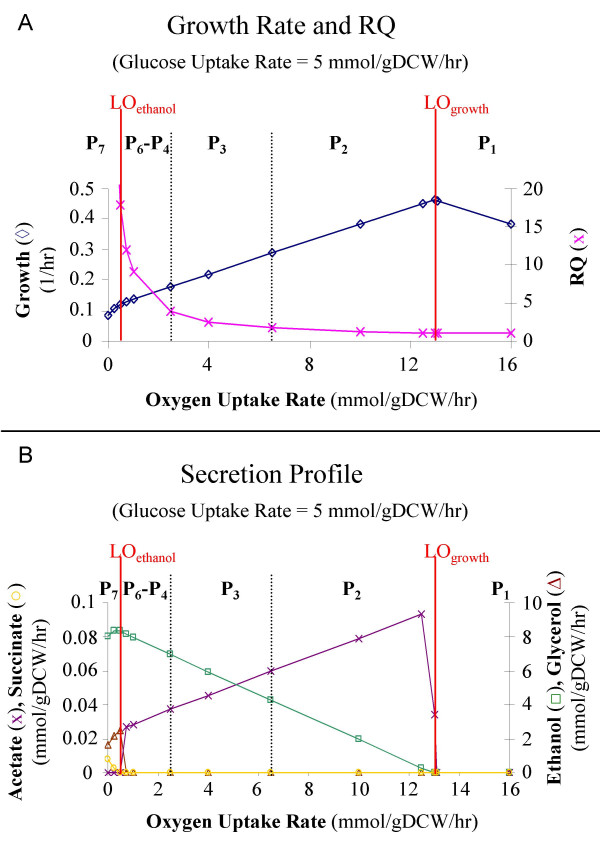
**Simulation of metabolic behavior for optimal cellular growth as a function of oxygen availability, ranging from completely anaerobic fermentation to completely aerobic growth in *S. cerevisiae***. The range of oxygen uptake rates used in the simulations (orange line, Fig. 1) allows for the characterization of the PhPP's seven phases (P_1 _- P_7_) and two lines of optimality (LO_growth_, LO_ethanol_). **(a) **Growth rate and respiratory quotient (RQ). **(b) **Secretion profile for acetate, succinate, ethanol, and glycerol.

Computer simulations (Fig. 1b) were used to illustrate the change of metabolic phenotypes described by the yeast phase plane. For the simulations, we arbitrarily set glucose uptake rate to 5 mmol/gDCW/hr and varied the oxygen uptake rate from 0 to 20 mmol/gDCW/hr. This allowed us to study the influence of a single environmental variable on cellular metabolism. Small amounts of NH_3_, sulfate and phosphate were introduced for the biomass synthesis. During anaerobic conditions (OUR = 0, on the *x*-axis), the growth rate was low and the respiratory quotient (RQ) was infinite by definition (Fig. 2a). As the oxygen uptake rate increased to 13 mmol/gDCW/hr to reach LO_growth_, the growth rate increased to its maximum value and the respiratory quotient approached 1.06. Further increasing the oxygen uptake rate caused both the growth rate and respiratory quotient to decrease due to futile cycles in which a combination of two or more biochemical reactions resulted only in the hydrolysis of ATP or other high-energy compounds [33].

Metabolic by-product secretion profiles also were calculated with increasing oxygen uptake rates. Since alternative optimal solutions exist in the genome-scale metabolic flux models [34], a range of secretion rates can be found amongst all of the equivalent optimal solutions for a fixed point in the PhPP. Remarkably, there was less than 1% difference between the maximum and minimum allowable secretion rates for a fixed maximal growth rate; thus, only the maximum predicted secretion fluxes for ethanol, succinate, glycerol, and acetate are shown (Fig. 2b). During anaerobic fermentation, ethanol, glycerol, and succinate were produced. Maximum ethanol production occurred at an oxygen uptake rate of 0.5 mmol/gDCW/hr, a condition defining LO_ethanol_. Glycerol production ceased at this point. With a slight increase in oxygen uptake rate above LO_ethanol_, acetate began to be secreted but succinate secretion decreased to zero. Ethanol and acetate were no longer secreted once the oxygen uptake rate was equal to or greater than 13 mmol/gDCW/hr, a point on LO_growth _where the metabolic pathway utilization enables complete aerobic growth.

### Further characterization of oxidative-fermentative phases (P2 - P6)

Linear programming simulations generate parameters called shadow prices that can be used to evaluate how changes in metabolite availability affect the biomass formation [33]. Shadow price analysis was used to further characterize the oxidative-fermentative phases. A positive shadow price indicated that a metabolite was available in excess, meaning that a decrease in its availability would increase biomass synthesis, and a negative shadow price indicated that a metabolite was limiting such that increasing its availability would increase the biomass synthesis.

*In silico *gene deletions were also performed in order to determine which reactions were essential in each phase. Essential reactions were defined as those whose deletion resulted in no predicted growth (growth rate equal to zero). This approach was especially useful for interpreting the physiological differences between growth states in phases 2 – 6 since their phenotypes were indistinguishable in terms of their secretion profiles.

#### Phase 2

In phase 2, the ratio of oxygen uptake rate and glucose uptake rate (GUR) is lower than that on the line of optimality. As a result, the cell is oxygen limited and begins to ferment. Mitochondrial NAD+ is available in excess, meaning that the biomass synthesis would improve if its availability decreased. In order to maintain the cell's redox balance, the excess mitochondrial NAD+ must be reduced. This is done through the production of acetate and ethanol, which begin to be secreted in this phase. Thus it is the production of acetate and ethanol that makes the optimal growth rate less than that defined on the line of optimality.

#### Phase 3

As the ratio of oxygen and glucose uptake rates is further decreased, three lower glycolysis reactions (fructose bis-phosphate aldolase, triose phosphate dehydrogenase, and phosphoglycerate kinase) become essential for growth in phase 3. Although these deletions severely hinder growth in phase 2 (reducing the growth rate by 55%, 19%, and 19%, respectively), the simulated growth rate is non-zero so these reactions were not considered to be essential. However, these reactions are essential in subsequent phases as the oxygen uptake rate is further decreased. Due to the limited oxygen, more carbons "overflows" into the fermentation pathway while at the same time oxidative metabolism becomes less effective.

#### Phase 4

Shifting from phase 3 to phase 4, the pentose phosphate pathway is utilized to generate NADPH because not enough NADPH is produced through respiration at the lower oxygen uptake rate. The NADPH is then converted to NADH which is subsequently used for ATP production.

#### Phase 5

Further lowering the ratio of oxygen and glucose uptake rates restricts the cell's ability to produce pyruvate in phase 5. Yeast can no longer utilize the oxidative pathways because an insufficient amount of cytosolic NAD+ is produced. When comparing phases 4 and 5, all of the metabolites with shadow price sign changes were folate intermediates. These are important energy carriers that are directly linked to the availability of both cytosolic and mitochondrial NAD+ and NADP+.

#### Phase 6

As you enter phase 6, the acetate production is completely ceased. Ethanol is secreted as the only metabolic by-product to balance the redox potential of the cell.

### Growth experiments

**Figure 3:**
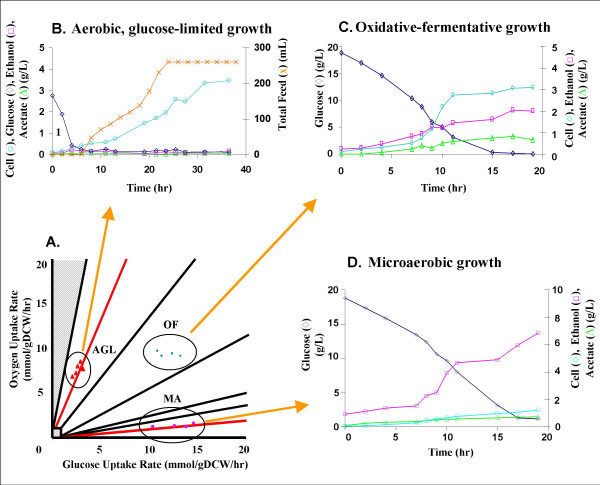
**Growth experiments shown on the PhPP. (a) **The three groups of experimental data displayed on the *S. cerevisiae *PhPP were used as an index for the time course profiles in panels (b), (c) and (d). **(b) **Aerobic glucose-limited growth controlled by fed-batch operation. **(c) **Oxidative-fermentative growth with unlimited glucose and oxygen availability. **(d) **Microaerobic growth with unlimited glucose and very low oxygen availability. The AGL (b) and MA (d) data sets are located on lines of optimality and as a result are stable metabolic states with only one degree of freedom (glucose for AGL and oxygen for MA). OF (c) is an unstable metabolic state with two degrees of freedom (glucose and oxygen), making it more difficult to control this type of growth condition. By perturbing the environmental conditions, cells in OF can be shifted to either AGL or MA (unpublished results).

Three groups of experiments were conducted under different growth conditions in the PhPP (Fig. 3a). These three conditions were:

• Aerobic, glucose-limited (AGL) growth experiments were conducted with a data acquisition and process control system. The dissolved oxygen (DO) level was maintained above 30% by sparging the compressed air into the CelliGen^® ^Plus bioreactor during the cell cultivation. The system was controlled at the respiratory quotient of 1.06 for optimal *S. cerevisiae *growth by RQ-stat feeding strategy to maintain the glucose concentration at a low, stable level (Fig. 3b). The cell concentration rose steadily with almost no acetate or ethanol production and most of the carbon was incorporated into the yeast biomass.

• Oxidative-fermentative (OF) batch growth experiments were carried out by allowing an essentially unlimited supply of oxygen and glucose. The DO level was maintained above 30% by sparging the compressed air into the shaker's flasks during the cell cultivation. Ethanol and acetate were accumulated in the aerated processes (Fig. 3c).

• Microaerobic (MA) batch cultivations with *S. cerevisiae *were performed at a low dissolved oxygen level. The experiments were performed in side-arm flasks (Fig. 3d) in which a small amount of air was allowed to diffuse into the flasks via the cotton filter on the side arms. The DO level was measured to be less than 5%. The initial glucose concentration and the limited oxygen supply resulted in high levels of ethanol and low levels of acetate.

#### Integration of experimental data and in silico predictions

The *S. cerevisiae *PhPP is a genome-scale model-based visualization platform which allows experimental data and simulation results to be displayed and compared. The three groups of batch and fed-batch experimental data are projected on Fig. 3a using the experimentally measured OUR and glucose uptake rates. These rates were then used as constraints in the computer simulations. Table 1 shows that the experimental observations and the *in silico *predictions are in good agreement.

**Table 1:** Comparison of *In Silico *Predictions and Experimental Measurements.

	Microaerobic fermentation		Oxidative-fermentative growth		Aerobic, glucose-limited growth	
	
	OUR = 1, GUR = 14		OUR = 9, GUR = 12		OUR = 8, GUR = 2.5	
	*In silico*	Experimental	*In silico*	Experimental	*In silico*	Experimental
Growth rate	0.33	0.31	0.53	0.51	0.22	0.20
Ethanol	21.29	20.08	11.98	11.07	0	0.16
Acetate	0.26	0.22	2.62	2.57	0	0.31

*Abbreviations: *oxygen uptake rate (OUR), glucose uptake rate (GUR)

*Units: *growth rate (1/hr), substrate uptake rates and metabolite production rates (mmol/gDCW/hr)

## Discussion

In this study, the *S. cerevisiae *genome-scale metabolic network constructed by Forster *et al. *[13] was used to generate a PhPP [9] that describes yeast's metabolic states at various levels of glucose and oxygen availability (Fig. 1). Examination of the *S. cerevisiae *PhPP has led to clear interpretation and prediction of its metabolic capabilities. First, only a few distinct optimal *S. cerevisiae *growth phenotypes were found *in silico*, and these phenotypes correspond to well-defined phases of the PhPP. Second, two lines of optimality were identified in yeast's PhPP: LO_growth_, which represents optimal biomass production during aerobic, glucose-limited growth, and LO_ethanol_, which corresponds to both maximal ethanol production and optimal growth during microaerobic conditions. The predictions of *S. cerevisiae*'s PhPP and genome-scale model were compared to independent experimental data. The results showed that the agreement between the computed and observed growth rates, uptake rates, and secretion rates was close to the measured values or within the experimental error, and qualitatively the predictions agreed with published literature.

Analysis of experimental data within the PhPP formalism suggests that yeast has only a few primary phenotypes, designated by the various phases. In P_1_, the oxygen supply is sufficient for growth by aerobic respiration, resulting in carbon dioxide as the sole by-product. Phases P_2_-P_6 _correspond to states of oxidative-fermentative growth, which is characterized by secretion of oxidative and fermentative metabolic by-products, *i.e.*, acetate and ethanol, respectively. These states are highly similar since the phases are essentially co-planar in the 3-dimensional PhPP (Fig. 1a). The secretion profile (Fig. 2b) does not show any phenotypic differences between phases P_2 _- P_6_. However, through the use of shadow price analysis and *in silico *gene deletions, distinct pathway utilization patterns could be found for each phase. Finally, P_7 _represents microaerobic conditions. In this environment, yeast grows primarily by fermentation and secretes ethanol, glycerol, and succinate (Fig. 2b). This limited range of metabolic states is strikingly different from that found for *E. coli*, whose glucose-oxygen PhPP has five distinct optimal *in silico *phenotypes [32]. Comparison of the simulation results generated by the *E. coli *[35] and *S. cerevisiae *[19] models indicates that *E. coli*'s metabolic by-product secretion patterns are more sensitive to the OUR variation than those of *S. cerevisiae*. Moreover, computer simulations show that when the OUR is lower than 7 mmol/gDCW/hr at a glucose uptake rate of 5 mmol/gDCW/hr, the TCA cycle in E. coli is broken into two branched pathways, one operating as a reductive pathway reversing the usual sequence from succinate to oxaloacetate and the other continuing to operate oxidatively to convert oxaloacetate to α-ketoglutarate. For *S. cerevisiae, *the pathway still functions as a cycle even when the OUR is as low as 1 mmol/gDCW/hr. Thus, it can be concluded by the comparison of *E. coli *and *S. cerevisiae *metabolic networks that yeast appears to be more robust to environmental perturbations. Furthermore, we predict that yeast secretes fewer metabolic by-products under these conditions, suggesting that its metabolism is more efficient than that of *E. coli*. This may represent a universal difference in how prokaryotes and eukaryotes respond to shifts in environmental parameters.

Another feature that distinguishes the *S. cerevisiae *PhPP from the *E. coli *PhPP is the existence of two definable lines of optimality. The conditions that define LO_growth _are similar to those that define *E. coli*'s sole line of optimality, *i.e. *they both represent the relationship between the glucose and oxygen uptake rates that results in optimal growth rate. Analysis of yeast's PhPP suggests that at a specific ratio of glucose and oxygen uptake rates glycerol production is halted and NADH is re-oxidized by maximal ethanol formation. This phenomenon, defined by LO_ethanol_, has been supported by many research reports in the literature [3,17,25]. For example, Cysewski and Wilke [37] found a sharp stimulation of the specific ethanol productivity at a very low but non-zero level of dissolved oxygen. Later studies showed that a value of 10 ppb of dissolved oxygen maximized ethanol production in yeast chemostat cultures [25]. Thus, LO_ethanol_, the second line of optimality predicted by the genome-scale model, is consistent with the experimental observations.

A useful application of the *S. cerevisiae *PhPP is to qualitatively classify yeast's metabolic state based on phenotypic observations made *in vivo *(Fig. 3). The metabolite concentration profile obtained from all of the experiments qualitatively agrees with the corresponding metabolic state predicted by the PhPP. For example, in growth conditions near LO_ethanol_, cells are expected to grow almost entirely by fermentation, with significant production of ethanol and lesser amounts of glycerol, acetate and succinate secretion. This phenotype is qualitatively similar to experimental observation, in which more ethanol is produced than acetate as shown in Fig. 3d. Points in the PhPP representative of the three data sets also were used to quantitatively predict yeast's metabolic phenotype (Table 1). Overall, the predictions are in good agreement with the experimental measurements. However, the predicted growth rates are slightly higher than the measured values. This difference may result from the model's prediction of optimal performance not reflecting suboptimal growth *in vivo*.

## Conclusions

The genome-scale metabolic networks developed for other microorganisms, namely *Escherichia coli*, *Haemophilus influenzae*, and *Helicobacter pylori*, have led to useful insights into substrate preferences, the effects of gene deletions, optimal growth patterns, outcomes of adaptive evolution, and shifts in expression profiles [22]. With the recent reconstruction of *S. cerevisiae*'s genome-scale metabolic network [19], these analytical techniques can now be applied to the first genome-scale model of an eukaryotic cell. By developing methods such as the PhPP to explore *in silico *the metabolic capabilities of microorganisms, we can generate new hypotheses as to how these organisms operate, and, more importantly, we can gain insight into the impact of individual cellular components on the organism as a whole.

## Methods

### Experimental methods

#### Strains and media

All cultures were grown at 30°C in SD medium [38] and supplemented with glucose (Sigma Chemical Co., St. Louis, MO) as appropriate for each phase of the experiment conducted. The *S. cerevisiae *strain *FY4 MATα *[39] was used in this study.

#### Growth and fermentation system

For experiments, 5 ml of overnight culture inoculated from single colonies grown on YPD agar was used to seed 50 ml of SD media pre-warmed to 30°C in a 250-ml Erlenmeyer flask, which was placed in a 30°C shaking incubator at 225 rpm for approximately 12 hours. This secondary seed was then used to inoculate either a 1.5-L Erlenmeyer flask with side arms for parallel batch fermentations or a 1.0-L bioreactor (CelliGen^® ^Plus, New Brunswick Scientific Co., Inc., Edison, NJ, USA). Cultures for aerobic, glucose-limited, fed-batch growth were initially grown in a batch mode, and a specific substrate limited after the culture reached particular biomass concentrations in each respective experimental condition. All batch culture experiments were performed in our multiple fermentation system which consists of acrylic enclosures filled with de-ionized water that can accommodate 32 cultures in parallel in batch operation mode at volumes ranging from 100 mL to 1500 mL capacity. We used this setup with either shaker's flasks as reactor vessels. A magnetic agitator (Bellco Glass, Inc., Vineland, NJ, USA) was used to continually mix flask contents at a speed of 225 rpm, and each flask was sealed with a rubber stopper containing apertures for probes, nutrient inlets/outlets, and sample harvesting. Temperature is strictly and uniformly controlled using a water circulator (model C10, Thermo Haake, Portsmouth, NH) with a temperature control module that drives a closed circuit of water to and from the controller to inlet and outlet drains on the water bath. Dissolved oxygen is measured and controlled using a polarographic electrode connected to DO meters/controllers. The fed-batch *S. cerevisiae *cultivations were automatically controlled in the 1-L bioreactor (CelliGen^® ^Plus, New Brunswick Scientific Co., Inc., Edison, NJ, USA). It has its own controllers for temperature, pH and dissolved oxygen (DO). A Pentium II computer (233 MHz processor, Microsoft Windows 98) equipped with an AT-MIO-16E-10 Analog Input computer interface board (National Instruments Corp., Austin, TX, USA) was used for data acquisition and process control for both the multiple fermentation system and CelliGen^® ^Plus bioreactor. Data from the batch and fed-batch cell cultures, including pH, temperature, and dissolved oxygen concentration were acquired through the interface board. The real-time graphical data acquisition and process control programs was written in LabVIEW 6.0 (National Instrument Corp., Austin TX). Media fed to the bioreactor was controlled by a feeding pump (Masterflex Computerized Drive 7550-90, Cole-Parmer Instrument Co., Chicago, IL, USA), with a RS 232 serial link accepting control signal from the computer, for fed-batch cell cultivation process. Acquisition of dissolved oxygen (DO) data was obtained with the aid of a respirometer [40] using a dissolved oxygen probe (Cole-Parmer Instrument Co., Chicago, IL, USA). For all the experiments, temperature was controlled at 30°C.

#### Determination of respiratory quotient (RQ)

The ratio of carbon dioxide evolution rate (CER) to oxygen uptake rate (OUR) has previously proven useful in inferring a lack of substrates in the growth medium and in the calculation of feeding rates [41]. For the fed-batch experiment, compressed air was fed into the bioreactor through a gas flowmeter (Manostat 125, New York, NY, USA), which was manually adjusted to a flow rate of 100 mL/min. The composition of exhaust gas from the bioreactor was measured using a gas analyzer (1440C Gas Analyzer, Servomex Co., Inc., Norwood, MA, USA) connected to the interface board to gauge exiting O_2 _and CO_2 _levels. Calculations for CER, OUR, and RQ were performed using the equations:









where O_2, in _and CO_2, in _and O_2, out _and CO_2, out _are the oxygen and carbon dioxide fractions in % v/v in the inlet air and exiting gas measurements, respectively, Q_in _and Q_out _are the air flow rates, and V_m _is the working volume of the bioreactor. When the estimated RQ reached 1.06, a peristaltic pump (Cole-Parmer) was utilized to begin feeding 10X concentrated growth medium into the bioreactor, and this quotient was maintained by an RQ-stat control strategy to limit the production of by-products or consumption of these by-products as an alternative energy source.

#### Sampling procedures

During cultivations, two separate 1-ml aliquot samples were taken at early, mid, and late log-phase from both the bioreactor and flasks. The first aliquot was used to determine cell density by measuring the optical density A_600_, A_420_, using a spectrophotometer (Beckman DU640, Beckman Coulter, Inc., Fullerton, CA, USA), and cell counts (Coulter Electronics Inc., Hialeah, FL, USA). The second aliquot was then filtered on a manifold containing a dry, pre-weighed, 0.2 μm pore-size filter to isolate a cell pellet, and was washed three times with 250 ml of sterile deionized water to ensure all salts were removed. After washing, the filters are placed in aluminum foil inside a 65°C incubator for 24 hours and subsequently weighed in an analytical balance to measure cell dry weight. The second aliquot was filtered through a 0.45 μm acrodisc syringe filter to separate cells from supernatant. The concentrations of metabolites in the supernatant such as glucose, acetate, ethanol, and glycerol were determined by using enzyme-based assay kits (glucose and acetate assay kit, Sigma Chemical Co., St. Louis, MO, USA; ethanol and glycerol assay kit, R-Biopharm, Inc., Marshall, MI, USA).

#### Growth rate, specific uptake/production rates and OUR_flask_

All specific growth rate curves were obtained by a linear regression of all data points within the exponential growth phase using the following formula *X *= *X*_*o*_*e*^*μt*^, where *X *is the cell concentration (gL^-1^), *X*_*o *_is the initial inoculum cell concentration (gL^-1^), *t *is the time of inoculation, and μ is the specific growth rate (1 hr^-1^). A minimum of ten optical density measurements were needed for the growth rate determination for both batch and fed-batch cultures.

The specific glucose uptake rate (GUR), ethanol and acetate formation rates, and OUR_flask _(OUR for the batch culture using flasks) were determined by fitting the dynamic mass balance equations for glucose, ethanol, acetate and DO measurements to the data points spanning the time period of the exponential cell growth phase. The specific uptake and production rates were then calculated by solving the dynamic mass balance equation within the culture medium using the following equation:



where *V *(L) is the culture volume, [S] (mM) is the substrate/product or DO concentration in the flask, *q *(mmole/g-dry weight/hr) is the substrate uptake rate or by-product formation rate or OUR_flask_, and *X(t) *(g-dry weight/L) is the biomass concentration at time = *t *(hr). + is for the by-product formation and - is for the substrate consumption. The solution to this equation was fitted to the experimental data points using the nonlinear estimation routine in Statistica (StatSoft Inc, Tulsa OK) or the solver in Microsoft Excel. All data, to be considered valid and included in the analysis, must have correlation coefficients of 0.95 or greater. The data that were generated in this way represented the "pseudo-steady-state" [42] of the batch or fed-batch cell culture, and thus suitable for the calculation of growth rate, specific uptake and production rates, and OUR_flask_.

### *In silico *calculations

#### Flux balance analysis and linear programming

A genome-scale *S. cerevisiae *metabolic network has been reconstructed [19]. The network includes 733 metabolites and 1175 metabolic reactions, which are compartmentalized between the cytosol and the mitochondria. In metabolic network analysis, the relationship between metabolite concentrations, ***x***, and reaction activities, ***v***, is described by the dynamic mass balance equation [43,44]:



where ***S ***is an *m × n *matrix of stoichiometric coefficients, ***x ***is an *m *× 1 vector of metabolite concentrations, and ***v ***is and *n *× 1 vector of reaction activities. Thus, the rows of **S **correspond to the internal metabolites and the columns represent the reactions in the network. Under steady-state conditions, the dynamic mass balance equation simplifies to:

*S *• *v *= 0     (Eq. 6)

Since the number of reactions is often greater than the number of metabolites, Eq. 6 is underdetermined and contains multiple solutions. One approach to solving Eq. 6 for microbial networks is to define a set of inputs and outputs that correspond to the growth conditions and use linear optimization to maximize the cell growth [35]. This approach has been successful in capturing the phenotypic behavior of *S. cerevisiae *for various growth conditions [21].

#### Phenotypic phase plane (PhPP) formulation

The *S. cerevisiae *PhPP displays optimal growth rates for all possible variations in two constraining environmental variables, such as the carbon substrate and oxygen uptake rates. In this study, the glucose uptake rate (x-axis) was allowed to vary from 0 to 20 mmol/gDCW/hr and the oxygen uptake rate (y-axis) ranged from 0.1 to 20 mmol/gDCW/hr. The oxygen uptake rate was not allowed to reach zero because anaerobic simulations required additional supplements to maintain cell growth (ergosterol and zymosterol). Linear programming was used to calculate the optimal growth rate for all points in the x-y plane. Growth rate values were then plotted as the z-axis to form the surface of a three-dimensional PhPP (Fig. 1a). A two-dimensional PhPP was formed by projecting the 3-D PhPP onto the x-y axis (Fig. 1b).

The phases of the PhPP were determined by the calculation of shadow prices [46], which describe the sensitivity of the objective function (Z) to changes in the availability of each metabolite:



where b_i _is the i^th ^metabolite and γ_i _is the i^th ^shadow price. Shadow prices were calculated for each point in the x-y plane during the linear programming simulations. By definition, phases were identified as regions of the PhPP in which all of the points have the same shadow prices. Lines of optimality, which represent the optimal ratio of glucose and oxygen uptake rates for maximal biomass synthesis, were also identified using shadow price analysis [45].

#### Shadow price analysis and *in silico* gene deletions

To obtain a physiological interpretation of the differences between the oxidative fermentative phases (phases 2–6), we analyzed how the shadow prices of key metabolites changed across the phase boundaries. Simulations were run at a fixed glucose uptake rate of 5 mmol/gDCW/hr and an oxygen uptake rate ranging from 1.5 to 15 mmol/gDCW/hr. The sign of the shadow price was used to identify whether a small change in the metabolite's availability would positively or negatively affect the objective value. According to the convention defined in [45], a negative shadow price indicates that a metabolite is limiting, *e.g. *the value of the objective function increases if the metabolite's net production increases or its net consumption decreases. Similarly, a positive shadow price indicates that a metabolite is available in excess and a shadow price equal to zero indicates that a change in the availability of the metabolite does not affect the objective value.

Phases 2 – 6 were also characterized by performing gene deletions *in silico *(as described in [20]). Single genes were deleted at a representative point within each phase to determine which reactions were essential for viability in that region.

#### Secretion profile calculations

The first step in generating the secretion profile was to calculate the optimal growth rate for a given glucose and oxygen uptake rate. For the simulations in Figures 2a and 2b, the glucose uptake rate was fixed at 5 mmol/gDCW/hr and the oxygen uptake rate varied from 0 to 16 mmol/gDCW/hr (ergosterol and zymosterol uptake rates of 5.92 × 10^-5 ^and 1.27 × 10^-4 ^mmol/gDCW/hr, respectively, were used for the calculation at OUR = 0). The simulations were then re-run with a fixed glucose uptake rate, oxygen uptake rate, and growth rate to determine the maximum and minimum secretion rates of each metabolite with a shadow price equal to zero.

## Authors' contributions

NCD calculated the phenotypic phase plane (Fig. 1), carried out the secretion profile simulations (Fig. 2), characterized the phases, calculated the flux predictions for Table 1, and drafted the manuscript. BOP conceived the study, participated in its design and coordination, and assisted with manuscript preparation. PF designed the study and conducted the growth experiments (Fig. 3, Table 1) and revised the manuscript. All authors have read and approved the final manuscript.
